# Medication effectiveness may not be the major reason for accepting cardiovascular preventive medication: A population-based survey

**DOI:** 10.1186/1472-6947-12-89

**Published:** 2012-08-09

**Authors:** Charlotte Gry Harmsen, Henrik Støvring, Dorte Ejg Jarbøl, Jørgen Nexøe, Dorte Gyrd-Hansen, Jesper Bo Nielsen, Adrian Edwards, Ivar Sønbø Kristiansen

**Affiliations:** 1 Research Unit of General Practice, University of Southern Denmark, Odense, Denmark; 2 Department of Public Health, Biostatistics, Aarhus University, Aarhus, Denmark; 3 Institute of Public Health, University of Southern Denmark, Odense, Denmark; 4 Department of Primary Care & Public Health, School of Medicine, Cardiff University, Wales, UK; 5 Department of Health Management and Health Economics, University of Oslo, Oslo, Norway

**Keywords:** Decision-making, Risk assessment, Risk communication, Preventive health services, Primary prevention, Cardiovascular disease, Health behavior

## Abstract

**Background:**

Shared decision-making and patients’ choice of interventions are areas of increasing importance, not least seen in the light of the fact that chronic conditions are increasing, interventions considered important for public health, and still non-acceptance of especially risk-reducing treatments of cardiovascular diseases (CVD) is prevalent. A better understanding of patients’ medication-taking behavior is needed and may be reached by studying the reasons why people accept or decline medication recommendations. The aim of this paper was to identify factors that may influence people’s decisions and reasoning for accepting or declining a cardiovascular preventive medication offer.

**Methods:**

From a random sample of 4,000 people aged 40–59 years in a Danish population, 1,169 participants were asked to imagine being at increased risk of cardiovascular disease and being offered a preventive medication. After receiving ‘complete’ information about effectiveness of the medication they were asked whether they would accept medication. Finally, they were asked about reasons for the decision.

**Results:**

A total of 725 (67%) of 1,082 participants accepted the medication offer. Even quite large effects of medication (up to 8 percentage points absolute risk reduction) had a smaller impact on acceptance to medication than personal experience with cardiovascular disease. Furthermore, increasing age of the participant and living with a partner were significantly associated with acceptance. Some 45% of the respondents accepting justified their choice as being for health reasons, and they were more likely to be women, live alone, have higher income and higher education levels. Among those who did not accept the medication offer, 56% indicated that they would rather prefer to change lifestyle.

**Conclusions:**

Medication effectiveness seems to have a moderate influence on people’s decisions to accept preventive medication, while factors such as personal experience with cardiovascular disease may have an equally strong or stronger influence, indicating that practitioners could do well to carefully identify the reasons for their patients’ treatment decisions.

## Background

Shared decision-making is based on the principle of patient autonomy [1,2] and practitioners of shared decision-making aim to assist patients in making decisions that are in line with their own values and judgments. If so, there is no universally “correct” decision, and the role of the practitioner is to inform and help the patient think about his or her values and opinions. It then becomes evident that studies evaluating why patients do or do not accept treatment proposals are necessary.

It is also well-known to healthcare personnel that patients do not always take medication as prescribed [3]. Adherence may be improved if patients’ decisions on whether to initiate medication are in accordance with their personal expectations and attitudes [4]. Expectations may be based upon knowledge, including the expected risk reduction resulting from the medication on offer [5]. Decisions concerning medication acceptance may also be influenced by attitudes based on personal life experiences, including educational attainment and earlier experiences with the disease in question, either personally or in the family. Earlier studies provide understanding of links between patients’ and doctors’ perceptions of the patient’s condition, including knowledge of the patient’s reasons for decisions made and subsequent adherence to medication [6,7]. Acknowledging the link between informed consent for medication and subsequent adherence, practitioners may therefore do well to try to gain more knowledge and understanding of their patients’ attitudes and fundamental health beliefs in order to improve communication, correct misinterpretations, and aid the patient in making optimal health decisions.

Lifestyle changes and medication are widely recommended in the prevention of cardiovascular disease (CVD). Recent studies from the US argue that aggressive and targeted interventions are needed to enhance provider and patient adherence to guidelines for CVD risk reduction [8,9]. Other studies define non-acceptance and non-adherence to prescribed medical treatment as unrecognized cardiovascular risk factors, resulting in a significant burden on health care resources [10,11]. In a Canadian study, the authors suggest policies such as education programmes for both the public and for healthcare professionals to avoid underuse of statins [12].

Although several preventive interventions for chronic diseases such as CVD exist, this population-based study focuses on cardiovascular preventive medication and evaluates communication of effectiveness of a cardiovascular preventive medication in the context of other personal characteristics that may also influence treatment acceptance. The target group was aged 40 to 59 years, and thus at some potential risk of cardiovascular disease due to age (with or without other risk factors), and able to relate to the hypothetical scenario in question. The aim of this study was to identify factors that may influence people’s decisions and subsequent reasoning for accepting or declining cardiovascular preventive medication.

## Methods

During October through November 2005, a random sample of 4,000 persons aged 40–59 (at 1 January 2005) in the municipality of Odense (approx. 185,000 inhabitants), Denmark, was invited for an interview. Invitees were drawn from the Central Office of Civil Registration (the CPR-Office) under the Ministry for Economic and Interior Affairs.

No information from medical records was obtained, but due to age the individuals belonged to a target group of potential candidates for cardiovascular prevention therapy. Non-responders were followed up with reminders by letter and/or telephone.

The interviews took place in a university building next to the university hospital just outside the city centre. Participants were informed that the interview would be about preventive health care. For their effort the respondents would receive a gift of either two bottles of wine or one box of chocolates. Interviews were conducted in 4-hour sessions in the afternoons over a period of six weeks. Each interview lasted for about 30 minutes.

The design of the interview guide was based on intensive discussions within the research group, two groups with lay persons and one group with health personnel, and ultimately on discussions with researchers from the Danish National Centre for Social Research. A group of trained interviewers were assigned to conduct the interviews. The interview guides were structured with pre-specified options.

All participants were asked questions on sociodemographic characteristics, comprising age, gender, marital status, family income, educational attainment and occupation. They were then asked to imagine being at increased risk of cardiovascular disease (the baseline risk) and being offered medication. No medication name was mentioned, but the features of the medication (effectiveness and side effects) resembled statins.

**Figure 1:**
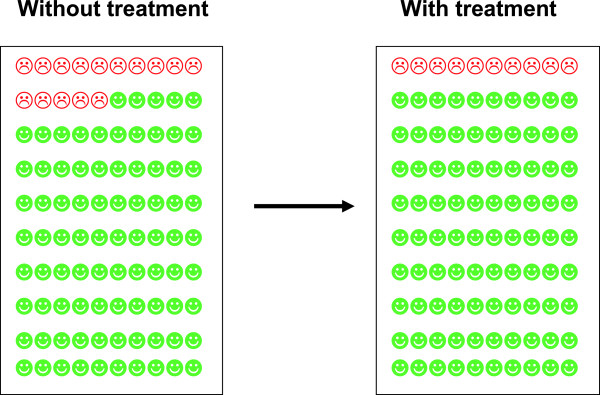
Example of pictorial presentation comprised in the ‘complete’ information.

Participants were allocated to four different levels of effectiveness, which regardless of information type was equivalent to an absolute risk reduction (ARR) of 2%, 4%, 5%, and 10%, and formed the bases for information about medication effectiveness. Even though they were initially presented with different formats of information (relative risk reduction, absolute risk reduction, number needed to treat, expected prolongation of life, as well as a pictorial presentation) [13] they were later all presented with the same ‘complete’ effectiveness information with a combination of all four formats for medication effectiveness presented, including a pictorial presentation (see Figure 1 presenting example of pictorial presentation). This “complete” information formed the basis of the decisions - to accept or not accept treatment - studied in this paper.

This paper reports on data from a study aimed at exploring the different ways of explaining risk reductions to lay people [13,14]. The study was powered to test hypotheses other than those tested here.

All the different effectiveness formats can be presented based on the same data, and none of the measures are more or less correct than the other, but all may affect decision; In order to minimize bias because of a specific format we presented “the full package”, and focused on the participants’ subsequent acceptance or rejection of cardiovascular preventive medication and the reasons for their decision. We refer to [13,14] for detailed description of the underlying design, including details of the risk information formats, interview guide and information cards.

While the initially presented risk format led to different acceptance rates [13], these differences were largely removed when participants had been presented with the final, “complete” information. We have therefore not allowed for an effect of the baseline risk and initial risk format, since all outcomes studied here are obtained after the final information had been provided.

We constructed the response options based on the Health Belief Model [15]. For participants who accepted medication, the options were: “For health reasons”, “Trust in my GP” or “Responsibility towards my family”. For participants who declined medication, the options were: “Too small effect of the medication”, “Wish to avoid side-effects”, “Do not want the extra expense”, “Dislike taking medication”, “Find the information difficult to understand”, “Prefer to change lifestyle”, and “General disbelief in effectiveness of medication”. For each of the above, participants were asked to pick the most important reason for the choice made. Finally, all participants were asked about their personal and family history of hypercholesterolemia, CVD or stroke, and asked four questions to capture their numeracy skills.

According to the Act on a Biomedical Research Ethics Committee System the project was not a biomedical research project and therefore did not need the ethic committee’s approval. The study was approved by the Danish Data Protection Agency.

### Statistics

The basic response variable was “acceptance of medication” on a binary scale (yes/no). To identify possible associations between participants’ characteristics and medication-taking behavior we first performed simple and multiple logistic regression modeling in Stata with acceptance of medication as the dependent variable. As explanatory variables we used medication effectiveness (ARR in percent), age, gender, duration of education, household income, numeracy skills, living with a partner, personal experience with cardiovascular disease or risk factors as presence of one or more of the following conditions: previous stroke or heart attack, hypercholesterolemia, or hypertension, and whether the participant had experienced cardiovascular disease in the family or not. The variables medication effectiveness, age and household income were used as continuous covariates, i.e. the corresponding odds ratio represents the estimated relative increase in odds due to a one unit increase in the covariate (percentage point, year, 100,000 DKK, respectively. US$1.00 = DKK5.90). The other variables were binary covariates.

In addition, we performed subgroup logistic regression analyses with people’s reasons for accepting medication as response variables. The analyses included separate logistic regression analyses for each of the three different response variables “For health reasons”, “Trust in my GP”, and “Responsibility towards the family”. Secondly, we performed separate subgroup logistic analyses of reasons for declining medication with each of the response variables “Too small effect of the medication”, “Wish to avoid side-effects”, “Dislike taking medicine”, and “Rather change lifestyle”. The options “Do not want the extra expense”, “Find the information difficult to understand” and “General disbelief in effectiveness of medication” were excluded from the analysis due to low frequency (1, 2, and 1 observations, respectively). As explanatory variables for the subgroup analyses we used medication effectiveness (ARR in percent), age, gender, duration of education, household income, numeracy skills, living with a partner, personal experience with cardiovascular disease or risk factors as presence of one or more of the conditions: previous stroke or heart attack, hypercholesterolemia or hypertension, and whether the participant had experience with cardiovascular disease in the family or not. The variables were chosen a priori from considerations based on expectations and knowledge within the field. For all estimates we report odds ratios (OR), and (95% confidence intervals). We chose logistic regression modeling because the outcome variable “acceptance of medication” is binary. We chose independent variables that would be plausible predictors of the outcome. We used non-parametric smoothing to assess linearity of the response variable on the log-odds scale with respect to continuous covariates. To build the model we had a priori established a list of covariates to include, but used the size of their estimated standard errors relative to the estimated effect size to judge both their impact and statistical significance. This was the case for both continuous and categorical covariates.

## Results

**Figure 2:**
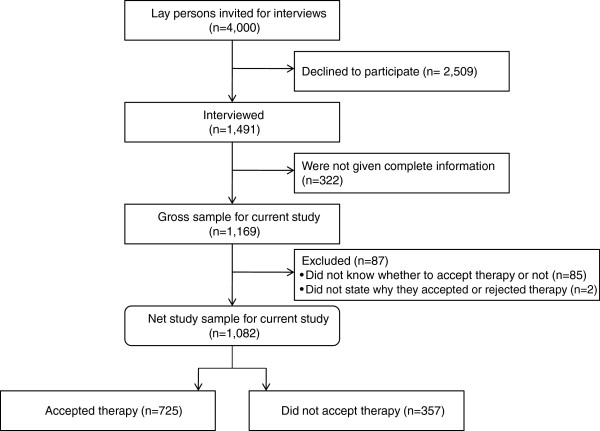
Participation flowchart.

Among the 4,000 invited, 1,169 persons participated (29.2%) and were given the ‘complete’ effectiveness information used for the present study. Because we aimed at exploring motives for accepting or declining the offered medication, the analyses did not include non-responders to the question as to whether or not they would accept the medication offer (n = 85). We assumed that these individuals could not make the decision whether to accept or not and thus were unable to state decision motives. Two individuals had not stated why they had accepted or declined the medication offer, and were likewise excluded from further analyses. Hence 1,082 participants were included for analyses (Figure 2).

Baseline characteristics of the 1,082 participants and participants grouped into those accepting to medication, respectively those declining, are reported in Table 1.

**Table 1:** Baseline characteristics of the participants according to acceptance or rejection of medication, and overall

	**Total**	**Acceptance of medication**	**Rejection of medication**
	**(n = 1,082)**	**(n = 725)**	**(n = 357)**
Covariate			
Age (yr), median (10–90 percentile)	51 (43;59)	52 (43;59)	50 (42;58)
Female gender	606 (56)	399 (55)	207 (58)
Short education (<10 years)*	395 (37)	273 (38)	122 (35)
Married or living w/ partner	839 (78)	577 (80)	262 (73)
Personal experience w/ cardiovascular disease	404 (37)	309 (43)	95 (27)
Family w/ known cardiovascular disease	441 (42)	305 (43)	136 (39)
Numeracy skills (less than 2 correct answers)	214 (20)	151 (21)	63 (18)
Household income	500-599	500-599	500-599
(1,000 DKK), median (10–90 percentile)	(200–299;800–899)	(200–299;800–899)	(200–299;800–899)

*Short education is defined as education level below lower secondary school including no specialist training or higher academic education.

Values are numbers (%) unless stated otherwise.

In total 725 (67%) respondents indicated that they would accept the medication offer. Table 2 shows crude and adjusted OR for variables potentially associated with acceptance of the medication offer.

**Table 2:** Logistic regression analysis of acceptance of medication offer (0 = accepting medication, 1 = declining medication)

**Independent variables**	**N**	**Crude OR (95% CI)**	Adjusted** OR (95% CI) (n = 981***)
Age (per year)	1,082	1.04 (1.02;1.07)	1.03 (1.00;1.05)
Female gender (ref. male)	606	0.89 (0.69;1.15)	0.87 (0.66;1.16)
Short education (<10 years) (ref. >10 years)*	395	1.17 (0.90;1.53)	1.09 (0.81;1.48)
Married or living w/ partner (ref. living alone)	839	1.42 (1.05;1.91)	1.56 (1.05;2.32)
Personal experience w/ cardiovascular disease (ref. no experience)	404	2.05 (1.56;2.71)	1.89 (1.40;2.55)
Family w/ known cardiovascular disease (ref. no family disease)	441	1.19 (0.92;1.55)	1.05 (0.80;1.39)
Numeracy skills (< 2 correct answers) (ref. >2 correct answers)	214	1.23 (0.89;1.70)	0.98 (0.68;1.42)
Household income (per 100,000 DKK)	1,014	0.98 (0.92;1.03)	0.94 (0.87;1.01)
Extent of medication effectiveness (per% point)	1,082	1.08 (1.03;1.13)	1.08 (1.03;1.14)

*Short education is defined as education level below lower secondary school including no specialist training or higher academic education.

** Adjusted OR accounting simultaneously for all 9 independent variables.

*** In total 101 missing observations due to a missing value for one or more independent variables.

Effect size of medication and personal experience with cardiovascular disease were both associated with acceptance of the medication offer, the effect of personal experience with disease equating an 8 percentage point increase in effect size (measured as ARR) offered in the multivariate model. Significant associations were also found between acceptance of the medication offer and increasing age of the participant as well as living with a partner.

Among those accepting medication, the main reasons were stated as “For health reasons” (46%), “Responsibility towards the family” (31%), “Trust in my GP” (18%) and “Other reasons” (4%). Answering “For health reasons” was associated with female gender, longer education, and living alone; answering “Trust in my GP” was associated with increasing age, living with a partner, and lower income; and answering “Responsibility towards the family” was associated with male gender and short education (Table 3).

**Table 3:** Logistic regression analysis for each of the main reasons for accepting medication

**Factors**	Adjusted** OR (95% CI)		
	**“For health reasons”**	**“Trust in the GP”**	**“Responsibility towards the family”**
	(n = 332/656***)	(n = 134/656***)	(n = 228/656***)
Age (per year)	0.99 (0.96;1.02)	1.05 (1.01;1.09)	0.98 (0.95;1.01)
Female gender (ref. male)	1.55 (1.12;2.15)	0.86 (0.57;1.31)	0.63 (0.45;0.89)
Short education (<10 years)* (ref. >10 years)	0.41 (0.29;0.59)	1.23 (0.83;1.95)	2.17 (1.50;3.13)
Married or living w/ partner (ref. living alone)	0.57 (0.35;0.91)	2.00 (1.10;3.63)	1.12 (0.67;1.89)
Personal experience w/ cardiovascular disease (ref. no experience)	0.95 (0.68;1.32)	1.41 (0.93;2.14)	0.85 (0.60;1.22)
Family w/ known cardiovascular disease (ref. no family disease)	1.00 (0.72;1.39)	0.78 (0.52;1.19)	1.03 (0.73;1.45)
Numeracy skills (< 2 correct answers) (ref. >2 correct answers)	0.93 (0.61;1.42)	1.06 (0.64;1.76)	1.14 (0.74;1.78)
Household income (per 100,000 DKK)	1.05 (0.96;1.15)	0.80 (0.71;0.90)	1.84 (0.98;1.19)
Extent of medication effectiveness (per% point)	0.97 (0.92;1.03)	0.99 (0.92;1.06)	1.04 (0.98;1.10)

*Short education is defined as education level below lower secondary school including no specialist training or higher academic education.

** Adjusted OR accounting simultaneously for all 9 independent variables.

*** In total 69 missing observations due to a missing value for one or more independent variables.

Among the 357 persons who declined the medication offer, the main reasons stated were “Prefer to change lifestyle” (58%), “Wish to avoid the side-effects” (19%), “Too small effect” (13%), “Dislike taking medicine” (5%), “Find the information difficult to understand” (1%), “Do not want the extra expense” (<1%), “General disbelief in effectiveness of medication” (0.3%), and “Other reasons” (4%).

Declining medication and justifying it with “Preference to change lifestyle” was associated with higher medication effectiveness, whereas the justification “Too small effect” was associated with lower medication effectiveness; answering “Wish to avoid the side-effects” was associated with short education. No significant associations were found with the reason “Dislike taking medicine” (Table 4).

**Table 4:** Logistic regression analysis for each of the main reasons for declining medication

**Factors**	Adjusted** OR (95% CI)			
	**“Too small effect”**	**“Want to avoid side-effects”**	**“Dislike taking medication”**	**“Rather change lifestyle”**
	(n = 46/324***)	(n = 67/324***)	(n = 17/324***)	(n = 207/324***)
Age (per year)	1.00 (0.94;1.06)	0.98 (0.93;1.03)	1.05 (0.95;1.16)	1.00 (0.96;1.04)
Female gender (ref. male)	1.19 (0.58;2.42)	0.60 (0.33;1.07)	1.46 (0.45;4.68)	1.40 (0.88;2.23)
Short education (<10 years)* (ref. >10 years)	1.03 (0.58;2.42)	1.89 1.03;3.47)	1.86 (0.60;5.76)	0.64 (0.39;1.06)
Married or living w/ partner (ref. living alone)	0.77 (0.30;2.01)	1.49 (0.64;3.47)	1.31 (0.34;5.05)	0.77 (0.40;1.48)
Personal experience w/ cardiovascular disease (ref. no experience)	0.72 (0.31;1.70)	0.98 (0.50;1.94)	0.83 (0.23;2.99)	1.18 (0.69;2.02)
Family w/ known cardiovascular disease (ref. no family disease)	0.98 (0.48;2.00)	0.86 (0.47;1.57)	0.53 (0.16;1.80)	1.34 (0.83;2.15)
Numeracy skills (< 2 correct answers) (ref. > 2 correct answers)	1.60 (0.66;3.90)	0.80 (0.35;1.82)	2.78 (0.86;8.96)	0.85 (0.45;1.58)
Household income (per 100,000 DKK)	0.99 (0.83;1.20)	0.95 (0.81;1.10)	0.75 (0.55;1.03)	1.05 (0.93;1.18)
Extent of medication effectiveness (per% point)	0.78 (0.66;0.93)	0.94 (0.84;1.06)	0.97 (0.80;1.19)	1.10 (1.01;1.20)

*Short education is defined as education level below lower secondary school including no specialist training or higher academic education.

** Adjusted OR accounting simultaneously for all 9 independent variables.

*** In total 33 missing observations due to a missing value for one or more independent variables.

We tested for colinearity, and the highest correlation was observed between “Household income” and “Age” (r = −0.11). Furthermore, we tested for interactions but found none.

For all regression analyses, coefficient estimates were relatively insensitive to adjustment for other covariates.

## Discussion

### Summary of main findings

A total of 67% of participants indicated that they would decline the medication offer after receiving information on medication effectiveness. Effectiveness of the intervention and personal experience with cardiovascular disease were both associated with acceptance of the medication offer – the effect of personal experience with disease equated an 8 percentage point increase in effect size (measured as ARR) offered in the multivariate model.

### Strengths and limitations of the study

Despite incentives to improve participant rates, the overall participation rate was low. The response rates for individual items among participants were, however, generally very high, which justifies the complete case approach of the data analysis.

Compared to the background population, a higher proportion of participants were women, the participants had an average annual household income below the national average of this age group, but had longer education than the background population of this age group [13]. Thus caution is required in generalizing to a wider section of the Danish population, or to other countries.

The findings of the present study were based on a large survey among lay people in a hypothetical scenario. While trying to resemble real life, and a decision to which people in this age range could relate, the findings will be but an estimate of reality and real life decision-making. On the other hand, this setting allowed us to provide participants with more detailed, nuanced and ‘complete’ information than in most clinical settings, but caution is also required in generalizing from hypothetical to real clinical decisions [16].

### Comparison with existing literature

The results of this study indicate that the medication effect seems to have a moderate influence on people’s decisions to accept preventive medication offers, while other factors such as a personal experience with cardiovascular disease may have an equally strong or stronger influence on people’s decisions.

In agreement with other studies [17] this study implies that people with personal experience of cardiovascular disease have an increased tendency to accept cardiovascular preventive medication. Experience does, however, not appear to influence the reasons for either accepting or declining the medication offer. It is worth noting that it was specifically personal experience with cardiovascular disease themselves, and not among family members, which appeared to be significantly associated with acceptance.

### Implications for clinical practice and future research

The findings of the study suggest that in real life doctor-patient consultations, information on treatment effectiveness may have an impact on medication acceptance, but that previous personal experience with illness, at least with cardiovascular disease, should not be ignored when trying to capture real patients’ medication-taking attitudes, values and intentions and to achieve shared decision-making. Also, cultural and language factors may play a role, but unfortunately we have no data, here.

These findings may have implications for practitioners in routine preventive consultations with their patients. To many practitioners the predominant reason for recommending medical treatment is the effectiveness of the drug. However, lay people may not be experienced in evaluating effectiveness information and they may make decisions based on heuristics. Often this may be based on a ‘single most important reason’ [18]. If patients’ primary concerns are indeed not the effectiveness of a possible medical treatment, understanding their underlying reasons for decision-making is important, and a refocus on the communication between patient and doctor may be warranted. Practitioners ought not to focus solely on communicating effectiveness to their patients, but also on patient characteristics, including discussing values, expectations, and previous experiences with the individual patient [19]. Early identification of these beliefs about medication may be important elements in counseling and informing patients, as well as being aware of the subtle contextual factors that may trigger intuitive and emotional decision processes that affect acceptance of a given medication.

There was some evidence in this study of poor numeracy affecting reasons for declining medication (‘dislike taking medicine’). The fact that effectiveness information plays only a minor role in patients’ decision-making may be due to lack of understanding of the given effectiveness information. If this is also the case among real patients making decisions about actual treatments, then further effort should be made to help patients and perhaps doctors as well in understanding basic statistics [20]. As noted above regarding cautions in generalisability, further research is warranted to explore medication-taking intentions and associated patient characteristics in real life, with real patients confronted with actual risk and effectiveness information and having to make shared decisions with their doctors or nurses.

## Conclusions

The level of intervention effectiveness seems to have a moderate influence on people’s decisions about preventive medication while other factors may have an equally strong or stronger influence. The findings suggest that doctors may do well to discuss the reasons for treatment decisions with their patients in order to share decision-making, optimize decisions and perhaps improve adherence.

## Competing interests

The authors declare that they have no competing interests.

## Authors' contributions

CGH did the principal interpretation of data and drafting of the manuscript. HS performed the statistical analyses and together with JN, ISK, DGH and JBN conceived the idea of the study, participated in its design and coordination. All authors contributed to the interpretation of data and to the writing and revising of the manuscript. All authors have read and approved the final manuscript.

## Pre-publication history

The pre-publication history for this paper can be accessed here:

http://www.biomedcentral.com/1472-6947/12/89/prepub
